# Thromboembolic prophylaxis in childhood idiopathic nephrotic syndrome. From pathophysiology to clinical management

**DOI:** 10.3389/fped.2026.1933209

**Published:** 2026-09-22

**Authors:** Tim Ulinski, Bilal Aoun

**Affiliations:** 1 Pediatric Nephrology Department, Trousseau Hospital, AP-HP.Sorbonne Université, Paris, France; 2 Faculty of Medicine, Sorbonne Université, Paris, France

**Keywords:** childhood, immunosuppression, LMWH (low molecular weight heparin), nephrotic syndrome, prophylaxis, steroids, thromboembolic prophylaxis, thrombosis

## Abstract

Thromboembolic complications remain among the most serious complications of childhood idiopathic nephrotic syndrome (INS). Although their incidence is considerably lower than in adults, venous thromboembolism continues to cause substantial morbidity and occasional mortality, including pulmonary embolism, cerebral venous sinus thrombosis, and extensive deep venous thrombosis. Preventive management remains controversial because most affected children may not develop thrombosis, whereas pharmacological prophylaxis exposes many low-risk patients to potential overtreatment. Current recommendations are largely based on retrospective studies, expert opinions, and extrapolation from adult nephrotic syndrome, while prospective pediatric evidence remains scarce. This review examines the contemporary understanding of thrombogenesis in childhood INS, emphasizing that hypercoagulability extends beyond urinary antithrombin loss to involve hepatic overproduction of procoagulant factors, impaired fibrinolysis, platelet activation, endothelial dysfunction, and hemoconcentration. We also discuss the possibility that historical thrombotic risk may have been amplified by prolonged corticosteroid exposure and consider whether the widespread adoption of steroid-sparing immunosuppressive therapies and earlier relapse detection may have modified the epidemiology of thromboembolism in the modern therapeutic era. Current preventive strategies—including no routine prophylaxis, biomarker-guided individualized approaches, aspirin, and prophylactic low-molecular-weight heparin (LMWH)—are critically reviewed. We argue that increasingly complex biomarker-based algorithms have not demonstrated clear clinical superiority over simple, pragmatic decision-making. Severe hypoalbuminemia provides a pragmatic surrogate of nephrotic severity and may therefore help identify children in whom the overall prothrombotic state is likely to be substantial. We are not proposing albumin <20 g/L as a validated predictor of thrombosis. We are proposing it as a pragmatic clinical trigger for considering prophylaxis in a disease in which no validated predictive model currently exists. We propose a pragmatic risk-stratified approach in which routine pharmacological prophylaxis is unnecessary for early biochemical relapses with minimal clinical manifestations but should be strongly considered in children and adolescents with clinically significant active nephrotic syndrome associated with severe hypoalbuminemia (approximately <20 g/L), provided no major contraindications exist. Where feasible, LMWH appears to be the most appropriate prophylactic agent. Until prospective pediatric studies better define thrombotic risk and validate prediction models, practical consistency based on clinically meaningful risk thresholds may provide a more rational framework than theoretical precision for thromboprophylaxis in childhood INS.

## Introduction

Thromboembolic complications remain among the most feared complications of childhood idiopathic nephrotic syndrome (INS). Although substantially less frequent in children than in adults with nephrotic syndrome, thrombotic events continue to represent a source of major morbidity and occasional mortality (1–3). This creates a clinical dilemma: most children with INS may never experience a thromboembolic event, yet the few who do may present with life-threatening complications such as pulmonary embolism, cerebral venous sinus thrombosis, renal vein thrombosis, or extensive deep venous thrombosis (1–4).

Reported incidence rates in pediatric INS vary considerably depending on study design, patient selection, and event ascertainment, with most retrospective series suggesting clinically apparent thromboembolic event rates of approximately 1 to 5% (1, 5). Higher rates have been reported in selected high-risk populations, including children with congenital nephrotic syndrome, steroid-resistant disease, prolonged severe hypoalbuminemia, central venous catheters, or critical illness (1, 3, 5). These estimates likely underestimate the true burden, as systematic screening for asymptomatic thrombosis is rarely reported.

The clinical significance of thrombosis in childhood INS does not lie in absolute frequency but rather in severity. Even rare events strongly influence physicians’ behaviour, particularly when they are sudden, difficult to anticipate, and potentially life threatening. A single severe pulmonary embolism or cerebral venous sinus thrombosis may profoundly shape subsequent prescribing habits, often more than cohort incidence data (3, 4, 6, 7).

An additional challenge is that the epidemiology informing current practice may no longer fully reflect contemporary pediatric INS management. Much of the available literature originates from eras characterized by heavier cumulative corticosteroid exposure, more prolonged nephrotic activity, and more limited access to modern steroid sparing, immunomodulatory strategies (1, 4, 5, 8). Contemporary care increasingly includes levamisole, mycophenolate mofetil, calcineurin inhibitors, and anti-CD20 therapies, with the aim of reducing relapse frequency, cumulative proteinuria, and steroid toxicity (8). The use of cyclophosphamide has decreased with the availability of less toxic agents. Whether this therapeutic evolution has materially altered thrombotic risk remains uncertain.

This uncertainty is clinically relevant. If thrombotic risk in modern pediatric INS has declined substantially, routine pharmacologic prophylaxis may represent overtreatment. If severe thromboembolic events remain unpredictable despite improved disease control, absence of prophylaxis may expose children to preventable harm. Thus, the central question is whether our preventive strategies remain aligned with contemporary disease treatment and biology.

## Literature search and methodology

This narrative review was based on a literature search conducted primarily through PubMed. The search focused on publications addressing thrombotic risk, mechanisms of hypercoagulability, and thromboprophylaxis in children with nephrotic syndrome. Search terms included combinations of nephrotic syndrome, children/pediatric, thrombosis, thromboembolism, hypercoagulability, antithrombin, protein C, protein S, hypoalbuminemia, thromboprophylaxis, anticoagulation, low-molecular-weight heparin, and aspirin. Relevant systematic reviews, clinical guidelines, consensus statements, observational studies, and mechanistic and clinical studies were considered, with preference given to pediatric data. Because this article was conceived as a narrative review rather than a systematic review, the literature search was not performed according to a prospectively registered or predefined systematic review protocol, and no formal systematic screening, risk-of-bias assessment, or quantitative synthesis was undertaken. The available evidence was therefore synthesized narratively to develop a clinically oriented view on thrombotic risk assessment and thromboprophylaxis in pediatric nephrotic syndrome.

## Pathophysiology of thrombosis in childhood idiopathic nephrotic syndrome

### Beyond urinary antithrombin loss

The prothrombotic state of nephrotic syndrome has traditionally been attributed to urinary loss of endogenous anticoagulant proteins, particularly antithrombin 3 (AT-3), resulting in acquired hypercoagulability (3, 7, 9, 10). While fundamentally valid, this model is incomplete and current evidence does not support this view (11). Thrombosis in childhood INS reflects a multifactorial disturbance involving coagulation activation, impaired fibrinolysis, platelet dysfunction, endothelial activation, altered blood flow, and potentially treatment related contributors (12, 13).

### Loss of endogenous anticoagulant mechanisms

Urinary loss of AT-3 remains a central component of nephrotic thrombophilia (1, 9, 10). Reduced circulating AT-3 activity may impair physiological inhibition of thrombin and factor Xa generation, favouring venous thrombosis (1, 10). Urinary loss of protein S may further compromise endogenous anticoagulant pathways, while protein C alterations appear less consistent, likely reflecting compensatory hepatic synthesis and methodological variability (7, 9, 10). Although mechanistically intuitive, this model alone does not fully explain clinical thrombosis, suggesting that additional prothrombotic mechanisms are required (14).

### Hepatic procoagulant compensation

Hypoproteinemia triggers compensatory excessive hepatic protein synthesis to normalize albumin losses and by this activity multiple procoagulant factors are synthesized. Increased fibrinogen, factors V, VII, VIII, X, and von Willebrand factor have all been documented in nephrotic syndrome, collectively shifting hemostatic balance toward thrombogenesis (1, 13).

This compensatory hepatic response represents a major amplifier of nephrotic hypercoagulability.

### Impaired fibrinolysis

Nephrotic syndrome is associated with complex alterations in fibrinolysis that may favor persistence of fibrin thrombi. Urinary loss of plasminogen may reduce fibrinolytic capacity, while increased expression of plasminogen activator inhibitor-1 (PAI-1) may suppress plasminogen activation. In addition, *α*2-macroglobulin (*α*2M), which is typically increased in nephrotic syndrome, is a broad protease inhibitor capable of inhibiting plasmin and tissue-type and urokinase-type plasminogen activators, and may therefore further modulate fibrinolytic activity. However, the contribution of *α*2-antiplasmin (*α*2AP) appears more complex: reduced circulating *α*2AP with urinary loss has been reported in nephrotic syndrome, potentially favoring fibrinolysis, whereas increased *α*2AP has been associated with renal vein thrombosis in some patients. Thus, although recent functional studies support impaired clot lysis in nephrotic syndrome, the fibrinolytic phenotype likely reflects a dynamic balance between several opposing mechanisms rather than a uniform defect in fibrinolysis (1, 7, 12). Thrombosis in INS therefore reflects both enhanced clot formation and reduced clot breakdown.

### Platelet activation

Platelet activation provides an additional prothrombotic layer. Increased platelet aggregability, reactive thrombocytosis, and enhanced thromboxane generation have all been described during active nephrotic syndrome (1, 9, 12). Proposed mechanisms include hyperlipidemia, altered plasma protein composition, endothelial dysfunction, and corticosteroid exposure.

However, pediatric INS associated thrombosis is predominantly, but not exclusively, venous, where thrombin generation and fibrin formation likely play a more important role than platelet driven arterial mechanisms.

### Endothelial dysfunction

The vascular endothelium likely represents an underappreciated contributor to thrombophilia in INS (1, 13, 15, 16). Endothelial cells regulate anticoagulant surface properties, fibrinolysis, vascular tone, and platelet interactions. In active INS, increased circulating von Willebrand factor, oxidative stress, altered nitric oxide bioavailability, inflammatory signaling, and hyperlipidemia may collectively promote endothelial activation (13, 15).

This perspective broadens nephrotic thrombosis beyond a purely hematologic disorder toward a more integrated vascular pathophysiology.

### Hemoconcentration and altered rheology

Children with active INS frequently experience relative intravascular volume contraction despite marked edema (1, 7). Reduced oral intake, vomiting, infection, aggressive diuretic therapy, or albumin diuretic strategies may exacerbate this state. The resulting hemoconcentration increases blood viscosity, slows venous flow, and promotes thrombogenesis, particularly during clinically significant relapses (7, 15).

### Are glucocorticoids part of the problem?

An important and insufficiently explored question is whether part of the historical thrombotic burden in INS may have been treatment amplified.

Systemic corticosteroid exposure has been associated with increased venous thromboembolic risk in multiple non nephrotic settings, although confounding by underlying disease severity remains difficult to exclude (17). Proposed mechanisms include increased fibrinogen synthesis, elevated factor VIII and von Willebrand factor, impaired fibrinolysis through increased PAI-1 expression, enhanced platelet reactivity, and endothelial activation (17, 18).

In childhood INS, where repeated exposure to high corticosteroid doses has historically been common, these effects may be clinically relevant. This does not imply that corticosteroids are the principal driver of thrombosis, but raises the possibility that evolving steroid sparing strategies and lower steroid exposure for the first flare may have altered contemporary thrombotic risk more than currently appreciated.

### Why no single biomarker solves the problem

The multiplicity of pathogenic mechanisms helps explain why no single laboratory marker reliably predicts thrombosis in childhood INS (1, 7). Antithrombin activity, fibrinogen, D-dimer, platelet count, and other coagulation parameters each capture only isolated aspects of a complex prothrombotic phenotype.

By contrast, severe hypoalbuminemia may serve as a pragmatic surrogate of the overall severity of the nephrotic state, integrating the magnitude of protein loss and several of the physiological changes accompanying active nephrosis.

While biologically imperfect, such an integrative marker may prove more clinically useful than increasingly granular but operationally cumbersome laboratory refinement.

## Has thromboembolic epidemiology changed in the modern therapeutic era?

A central but insufficiently examined question is whether the thromboembolic risk historically associated with childhood INS remains representative of contemporary disease management. Current preventive strategies are largely informed by retrospective cohorts, case series, and expert experience accumulated over several decades, yet pediatric INS treatment has changed substantially during this period (1). Much of the literature informing current estimates of thromboembolic risk derives from therapeutic eras characterized by greater cumulative corticosteroid exposure and more prolonged nephrotic activity. These historical data remain relevant as a comparator, but their applicability to contemporary pediatric INS management is uncertain.

Historically, children with frequently relapsing or steroid dependent INS often accumulated prolonged exposure to high doses of corticosteroids, repeated episodes of sustained nephrotic relapse, and longer periods of active disease before effective immunomodulatory maintenance strategies became widely available. Contemporary management increasingly incorporates levamisole, mycophenolate mofetil, calcineurin inhibitors (mainly tacrolimus), and anti-CD20 therapies to reduce relapses, shortening nephrotic exposure, and minimizing cumulative corticosteroid toxicity (19).

These therapeutic changes could plausibly alter thrombotic risk through several mechanisms. Reduced cumulative nephrotic burden may shorten exposure to the most thrombogenic phases of disease, characterized by severe hypoalbuminemia, hemoconcentration, and systemic physiological disturbance (1, 15). Steroid minimization may represent an additional modifier if corticosteroids contribute directly to thrombogenicity (17, 18). Improved ambulatory monitoring with early relapse detection may further reduce time spent in advanced active nephrosis, particularly in experienced families managing relapsing disease (20–26).

At the same time, countervailing trends deserve consideration. Adolescents with INS may increasingly resemble adult nephrotic populations in thrombotic phenotype, while medically complex patients, central venous access, obesity, and prolonged steroid resistant or dependent disease may present high risk subgroups (1, 5).

The difficulty is that contemporary epidemiological clarity is lacking. Much of the literature informing current practice derives from therapeutic eras that differ considerably from nowadays pediatric nephrology, and prospective cohorts specifically designed to quantify thromboembolic risk in the modern steroid sparing era are lacking.

Importantly, this uncertainty should not be interpreted as evidence that thrombotic risk has become negligible. Serious and occasionally fatal thromboembolic events continue to occur, including pulmonary embolism and cerebral venous sinus thrombosis, and remain part of the collective clinical experience of pediatric nephrologists (3, 4, 6, 27). Thus, the relevant question is not whether thrombosis still occurs, but whether current preventive strategies are calibrated to contemporary rather than historical risk.

## Preventive strategies: highly individualized or pragmatic prevention

Preventive strategies for thromboembolism in childhood INS remain highly heterogeneous across pediatric nephrology centres, reflecting limited evidence, differing clinical philosophies, and variable healthcare infrastructure. The core dilemma is unavoidable: prophylaxis exposes many children who would never experience thrombosis, while withholding prophylaxis risks rare but potentially devastating events (Table 1).

**Table 1:** Proposed approach to thromboprophylaxis strategy according to clinical scenario in children with idiopathic nephrotic syndrome.

Clinical scenario	Proposed strategy
Early biochemical relapse, minimal edema, preserved condition	No routine prophylaxis, no systematic blood analysis
Moderate relapse without major high-risk features	Individualized assessment
Clinically significant active INS + albumin <20 g/L	Prophylactic LMWH
Previous thrombosis	Strong consideration for prophylaxis
Central venous catheter + active nephrosis	Earlier consideration of intervention
Adolescent with severe relapse	Earlier consideration of intervention

### No routine pharmacologic prophylaxis

A conservative strategy adopted by many centres is to avoid routine thromboprophylaxis, reserving anticoagulation for exceptional circumstances or established thrombosis (28, 29).

This approach is supported by the relatively low incidence of clinically apparent thromboembolism in unselected pediatric INS populations and by legitimate concerns regarding bleeding risk, treatment burden, and the practical challenges of prolonged anticoagulation in children.

Such an approach may be entirely reasonable in children with very early relapse recognition, minimal edema, preserved general condition, and rapid therapeutic response.

However, when applied broadly, it relies heavily on subjective clinical judgement, resulting in substantial variability among centres.

### Biomarker guided individualized prophylaxis

A more individualized strategy attempts to refine thrombosis risk assessment through laboratory markers such as antithrombin activity, fibrinogen, D-dimer, or broader coagulation profiles (1, 9, 10, 13, 29).

While conceptually attractive, this approach faces important practical limitations. Most biomarkers reflect only isolated dimensions of nephrotic thrombophilia, pediatric decision thresholds remain poorly standardized, and interpretation is often uncertain (1, 9, 10, 13, 29).

More importantly, their incremental clinical utility remains unclear. In a child with severe active nephrosis and profound hypoalbuminemia, biomarker refinement rarely alters the practical conclusion that thrombotic risk is elevated.

This does not preclude selective use in specific circumstances, but routine biomarker based prophylaxis algorithms risk adding complexity, cost, and inconsistency without clear evidence of improved outcomes.

### Inherited and acquired thrombophilia

Inherited thrombophilia may coexist with nephrotic syndrome and can be considered when the thrombotic event appears disproportionate to the severity or duration of nephrosis, occurs at an unusual site, is recurrent, or is associated with a strong family history of venous thromboembolism. Factor V Leiden and the prothrombin G20210A variant are among the most common inherited thrombophilias, while deficiencies of antithrombin, protein C, and protein S may also contribute to thrombotic risk. However, routine screening of all children with NS has not been shown to improve risk stratification or prophylactic management, and interpretation of protein C, protein S, and antithrombin levels during active nephrosis may be particularly challenging. Acquired thrombophilic conditions, particularly antiphospholipid antibodies in children with features suggestive of autoimmune disease, should likewise be considered when clinically appropriate. Thus, thrombophilia testing is best individualized according to the clinical context rather than performed systematically in all children with nephrotic syndrome (30, 31).

### Aspirin prophylaxis

Aspirin represents a pragmatic intermediate strategy used by some experienced pediatric nephrology teams, particularly where prolonged injectable anticoagulation is considered impractical (28).

Its advantages are clear: oral administration, familiarity, low cost, and avoidance of daily low molecular weight heparin (LMWH) injections and anticoagulation monitoring.

However, limitations should be acknowledged. Pediatric INS associated thrombosis is predominantly venous, where thrombin generation and fibrin formation likely play a greater role than platelet driven arterial mechanisms (1, 13). Aspirin may therefore address only a part of the prothrombotic process.

In addition, irreversible platelet inhibition complicates procedural planning, particularly when kidney biopsy or urgent invasive interventions are required, but some teams still perform kidney biopsies even if the patient is on aspirin treatment. The benefit-risk ration is not easy to assess.

Thus, while aspirin remains a pragmatic option in some settings, evidence supporting its efficacy for venous thromboprophylaxis in childhood INS remains limited (32, 33).

*Underlying disease and histopathology may further modify thrombotic risk.*

Congenital nephrotic syndrome and steroid-resistant nephrotic syndrome appear to carry a particularly high risk of thromboembolism, while focal segmental glomerulosclerosis (FSGS) is frequently represented among children who develop thrombotic events. Membranous nephropathy has also been associated with increased thrombotic risk, although pediatric data are very limited. Importantly, these associations should not be interpreted as evidence that histopathology alone determines the need for thromboprophylaxis. Rather, the underlying disease should be considered as one component of an integrated risk assessment, together with the severity and persistence of nephrosis, hypoalbuminemia, proteinuria, previous thrombosis, age, central venous access, infection, immobility, and other acquired or inherited risk factors (34).

### Systematic prophylactic anticoagulation in clearly defined high risk states

An alternative strategy is protocolized prophylactic anticoagulation in predefined high risk nephrotic states rather than unrestricted individualized decision making (1, 28).

In our view, this represents the most coherent approach in pediatric nephrology settings where anticoagulation can be safely implemented and monitored.

A child with trivial biochemical relapse detected through close home monitoring is biologically distinct from a child with clinically significant active nephrosis characterized by severe hypoalbuminemia, generalized edema, abdominal symptoms, reduced mobility, or impaired general condition.

For this reason, a pragmatic threshold based strategy appears preferable to either universal prophylaxis or fully discretionary individualized management.

In children and adolescents with clinically significant active INS and severe hypoalbuminemia (for example serum albumin <20 g/L), prophylactic anticoagulation appears justified in the absence of major contraindications.

No threshold is biologically exact, but clinical decision making frequently relies on pragmatic operational cut-offs. Serum albumin offers practical advantages as a rapidly available, reproducible, and integrative marker of nephrotic severity (8).

Where feasible, LMWH remains the most coherent prophylactic agent given its mechanistic alignment with predominantly venous thrombosis prevention and extensive pediatric clinical familiarity.

## Toward a pragmatic clinical approach

The absence of universally accepted pediatric thromboprophylaxis guidelines should not preclude practical structure (24, 30, 32). In routine care, clinicians must make rapid decisions despite imperfect evidence.

A pragmatic framework should be clinically applicable, biologically plausible, simple enough for reproducible implementation, and adaptable to differing healthcare systems.

Underlying disease etiology should also be considered. Children with congenital or genetically determined nephrotic syndromes, particularly those with severe or prolonged nephrosis, may represent a distinct high-risk population, although current evidence does not establish a specific histopathological or genetic subtype as an independent predictor of thrombosis. A personal or family history suggestive of hereditary thrombophilia may further support earlier intervention for prophylaxis or prompt targeted thrombophilia assessment.

### Low-risk situations

Routine pharmacologic prophylaxis may not be necessary in children with:

- very early biochemical relapse detected through close home monitoring;
- minimal or absent edema;
- preserved mobility and oral intake;
- stable general condition;
- serum albumin not yet severely reduced.

In such patients, anticipated exposure to severe thrombogenic physiology is likely brief, making routine anticoagulation difficult to justify (8, 11, 15).

### Intermediate-risk situations

A broad intermediate zone inevitably exists where management should remain individualized.

Relevant modifiers include:

- adolescence
- obesity
- previous thromboembolic events
- known thrombophilia
- central venous access
- infection
- dehydration or intravascular contraction
- prolonged relapse duration
- steroid-resistant nephrotic syndrome

The presence of these factors may reasonably support earlier considerationof prophylactic intervention.

### High-risk situations

Systematic prophylactic anticoagulation appears most justified in children and adolescents with:

- clinically significant active nephrotic syndrome;
- substantial edema;
- reduced mobility or impaired general condition;
- abdominal pain, vomiting, or systemic symptoms;
- severe hypoalbuminemia (e.g., < 20 g/L);
- no major contraindication to anticoagulation.

In these patients, prophylactic LMWH appears the most coherent preventive strategy where implementation is feasible.

### Special consideration: healthcare infrastructure

Pragmatic recommendations cannot be entirely separated from healthcare infrastructure.

A prophylactic strategy that is feasible in a tertiary pediatric nephrology center with rapid laboratory access, specialist monitoring, and anticoagulation expertise may be less realistic in resource constrained settings.

Thus, while general principles may be broadly applicable, implementation will inevitably vary across healthcare systems.

## Research priorities and unresolved questions

Despite longstanding clinical concern regarding thromboembolic complications in childhood INS, the evidence base guiding prophylactic decision-making remains limited. Much of current practice continues to rely on retrospective cohorts, case reports, institutional experience, and extrapolation from adult nephrotic syndrome rather than prospective and specific pediatric data.

Several key questions remain unsolved:

### Has thrombotic risk changed in the modern therapeutic era?

Contemporary care increasingly consists of modern steroid-sparing immunosuppressive strategies, among which anti-CD20 therapies have had a significant impact on INS management, but also earlier ambulatory relapse detection. All these points may reduce cumulative exposure to severe active nephrosis and high dose corticosteroids.

Whether these changes have translated into lower thromboembolic risk remains unknown. Prospective contemporary pediatric cohort data are urgently needed.

### Which children truly benefit from prophylaxis?

Although several risk factors are broadly recognized (including severe hypoalbuminemia, adolescence, prolonged active nephrosis, central venous access, previous thrombosis, infection, dehydration, and steroid resistant disease) their relative predictive value remains poorly quantified.

Validated pediatric risk prediction models are lacking, leaving clinicians to rely largely on expert judgement.

### Do biomarkers improve clinical decision-making?

Antithrombin activity, fibrinogen, D-dimer, thrombin generation assays, platelet function testing, and endothelial biomarkers may provide mechanistic insights, but their practical clinical utility remains uncertain.

The critical question is not biological plausibility, but whether such measurements meaningfully improve prophylactic decision-making beyond simpler clinical approaches.

### What is the optimal preventive strategy?

Even among clinicians favoring thromboprophylaxis, the optimal preventive agent remains uncertain. LMWH is mechanistically attractive for venous thrombosis prevention and familiar in pediatric practice, but imposes injection burden and monitoring challenges.

Aspirin offers greater simplicity but may incompletely address the dominant biology of nephrotic thrombosis. Direct oral anticoagulants may represent an interesting future option, but pediatric INS-specific evidence remains insufficient (35). Comparative prospective studies are needed, although the rarity of events will make conventional trial design challenging.

### Is part of the thrombotic burden treatment related?

The specific contribution of corticosteroid exposure to thrombotic risk in childhood INS remains poorly defined. If steroid exposure proves to meaningfully amplify thrombogenicity, evolving steroid-minimizing strategies may have implications not only for disease control but also for thromboprophylaxis paradigms.

### Towards a pragmatic rather than theoretical precision

Given the rarity of pediatric thromboembolic events, highly granular biomarker-based precision models may prove less clinically useful than pragmatic studies addressing operational questions.

For example:

- Does albumin threshold based prophylaxis reduce clinically meaningful events?
- Is aspirin an acceptable alternative to LMWH in selected pediatric settings?
- Which patient subgroups derive measurable benefit?

## Conclusion

Thromboembolic complications in childhood INS remain relatively rare. Severe events including pulmonary embolism and cerebral venous sinus thrombosis continue to shape pediatric nephrology practice disproportionally to their absolute incidence.

At the same time, current preventive strategies rely on an evidence base that is fragmented, largely retrospective, and partly rooted in therapeutic eras that may no longer fully reflect modern pediatric INS management.

Thrombotic risk in childhood INS is not uniform. Early biochemical relapse detected through close surveillance is biologically distinct from clinically significant active nephrosis with severe hypoalbuminemia, edema, and systemic clinical impact.

No biomarker perfectly predicts thrombosis, and no operational threshold is biologically exact. However, the absence of a threshold does not eliminate arbitrariness; it merely transfers arbitrariness to individual clinician judgement.

In our view, in pediatric nephrology settings where safe implementation is feasible, a pragmatic strategy based on systematic prophylactic anticoagulation for children and adolescents with clinically significant active INS and severe hypoalbuminemia represents the most coherent current approach, while lower-risk early relapses may reasonably be managed without routine pharmacologic prophylaxis.

The field may not require greater theoretical complexity, but clearer prospective evidence. Until then, pragmatic consistency may be preferable to potentially misleading precision.

## References

[1] Kerlin BA Ayoob R Smoyer WE. Epidemiology and pathophysiology of nephrotic syndrome-associated thromboembolic disease. Clin J Am Soc Nephrol. (2012) 7:513–20. 10.2215/CJN.10131011 22344511 PMC3302669

[2] Kerlin BA Haworth K Smoyer WE. Venous thromboembolism in pediatric nephrotic syndrome. Pediatr Nephrol. (2014) 29:989–97. 10.1007/s00467-013-2525-5 23812352 PMC6556227

[3] Suri D Ahluwalia J Saxena AK Sodhi KS Singh P Mittal BR, et al. Thromboembolic complications in childhood nephrotic syndrome: a clinical profile. Clin Exp Nephrol. (2014) 18:803–13. 10.1007/s10157-013-0917-2 24346593

[4] Ulinski T Guigonis V Baudet-Bonneville V Auber F Garcette K Bensman A. Mesenteric thrombosis causing short bowel syndrome in nephrotic syndrome. Pediatr Nephrol. (2003) 18:1295–7. 10.1007/s00467-003-1281-3 14564498

[5] Wang Z Tang H-Y Lin Q Li X-Z. Incidence of thromboembolism in children with primary nephrotic syndrome: a systematic review and metaanalysis. BMC Nephrol. (2023) 24:120. 10.1186/s12882-023-03160-y 37127615 PMC10150676

[6] Konopásek P Piteková B Krejčová V Zieg J. Cerebral sinovenous thrombosis in children with nephrotic syndrome: systematic review and one new case. Front Pediatr. (2023) 11:1207871. 10.3389/fped.2023.1207871 PMC10484110 37691772

[7] Llach F. Hypercoagulability, renal vein thrombosis, and other thrombotic complications of nephrotic syndrome. Kidney Int. (1985) 28:429–39. 10.1038/ki.1985.149 3906225

[8] Vivarelli M Gibson K Sinha A Boyer O. Childhood nephrotic syndrome. Lancet. (2023) 402:809–24. 10.1016/S0140-6736(23)01051-6 37659779

[9] Citak A Emre S Sâirin A Bilge I Nayır A. Hemostatic problems and thromboembolic complications in nephrotic children. Pediatr Nephrol. (2000) 14:138–42. 10.1007/s004670050029 10684364

[10] Mehls O Andrassy K Koderisch J Herzog U Ritz E. Hemostasis and thromboembolism in children with nephrotic syndrome: differences from adults. J Pediatr. (1987) 110:862–7. 10.1016/s0022-3476(87)80397-9 3585601

[11] Abdelghani E Waller AP Wolfgang KJ Stanek JR Parikh SV Rovin BH. Exploring the role of antithrombin in nephrotic syndrome-associated hypercoagulopathy: a multi-cohort study and meta-analysis. Clin J Am Soc Nephrol. (2023) 18:234–44. 10.2215/CJN.0000000000000047 36754010 PMC10103265

[12] Loscalzo J. Venous thrombosis in the nephrotic syndrome. N Engl J Med. (2013) 368:956–8. 10.1056/NEJMcibr1209459 23465106

[13] Singhal R Brimble KS. Thromboembolic complications in the nephrotic syndrome: pathophysiology and clinical management. Thromb Res. (2006) 118:397–407. 10.1016/j.thromres.2005.03.030 15990160

[14] Mykhaloiko IS Yatsyshyn RI Mykhaloiko IY. Natural anticoagulant deficiencies in patients with nephrotic syndrome: comparative evaluation of antithrombin iii, protein C and protein S and their association with hypercoagulability markers. Sci Pract J. (2026) 38(2):55–61. 10.21802/artm.2026.2.38.55

[15] Gigante A Barbano B Sardo L Martina P Gasperini M Labbadia R. Hypercoagulability and nephrotic syndrome. CVP. (2014) 12:512–7. 10.2174/157016111203140518172048 22724465

[16] Kelddal S Grove EL Duus CL Nygaard LB Kristensen T Mose FH. Alterations in coagulation and endothelial function in nephrotic syndrome: a multicenter, cross-sectional analysis. Kidney360. (2025) 6:1960–9. 10.34067/KID.0000000865 40478760 PMC12626668

[17] Johannesdottir SA Horváth-Puhó E Dekkers OM Cannegieter SC Jørgensen JOL Ehrenstein V. Use of glucocorticoids and risk of venous thromboembolism: a nationwide population-based case-control study. JAMA Intern Med. (2013) 173:743–52. 10.1001/jamainternmed.2013.122 23546607

[18] Van Zaane B Nur E Squizzato A Gerdes VEA Büller HR Dekkers OM. Systematic review on the effect of glucocorticoid use on procoagulant, anti-coagulant and fibrinolytic factors. JThromb Haemost. (2010) 8:2483–93. 10.1111/j.1538-7836.2010.04034.x 20735729

[19] Frangou E Kronbichler A Steiger S Bruchfeld A Caravaca-Fontán F Mirioglu S. The 2025 KDIGO guideline on the management of nephrotic syndrome in children: a comment of the European renal association immunonephrology working group. Clin Kidney J. (2026) 19:sfaf381. 10.1093/ckj/sfaf381 41522688 PMC12789865

[20] Basu B Sander A Roy B Preussler S Barua S Mahapatra TKS. Efficacy of rituximab vs tacrolimus in pediatric corticosteroid-dependent nephrotic syndrome: a randomized clinical trial. JAMA Pediatr. (2018) 172:757–64. 10.1001/jamapediatrics.2018.1323 29913001 PMC6142920

[21] Delbet J-D Hogan J Parmentier C Ulinski T Dossier C. Successful global anti-B-cell strategy with daratumumab in a patient with post-transplant nephrotic syndrome recurrence unresponsive to immunoadsorption and obinutuzumab. Pediatr Transplant. (2023) 27:e14544. 10.1111/petr.14544 37226549

[22] Dossier C Bonneric S Baudouin V Kwon T Prim B Cambier A. Obinutuzumab in frequently relapsing and steroid-dependent nephrotic syndrome in children. CJASN. (2023) 18:1555–62. 10.2215/CJN.0000000000000288 37678236 PMC10723910

[23] Dossier C Delbet J-D Boyer O Daoud P Mesples B Pellegrino B. Five-year outcome of children with idiopathic nephrotic syndrome: the NEPHROVIR population-based cohort study. Pediatr Nephrol. (2019) 34:671–8. 10.1007/s00467-018-4149-2 30552564

[24] Iijima K Sako M Kamei K Nozu K. Rituximab in steroid-sensitive nephrotic syndrome: lessons from clinical trials. Pediatr Nephrol. (2018) 33:1449–55. 10.1007/s00467-017-3746-9 28717938 PMC6061657

[25] Iijima K Sako M Nozu K Mori R Tuchida N Kamei K. Rituximab for childhood-onset, complicated, frequently relapsing nephrotic syndrome or steroid-dependent nephrotic syndrome: a multicentre, double-blind, randomised, placebo-controlled trial. Lancet. (2014) 384:1273–81. 10.1016/S0140-6736(14)60541-9 24965823

[26] Ravani P Bonanni A Rossi R Caridi G Ghiggeri GM. Anti-CD20 antibodies for idiopathic nephrotic syndrome in children. Clin J Am Soc Nephrol. (2016) 11:710–20. 10.2215/CJN.08500815 26585985 PMC4822672

[27] Stabouli S Chrysaidou K Kupferman JC Zafeiriou DI. Neurological complications in childhood nephrotic syndrome: a systematic review. Eur J Paediatr Neurol. (2019) 23:384–91. 10.1016/j.ejpn.2019.02.007 30837193

[28] Kidney Disease: Improving Global Outcomes (KDIGO) Glomerular Diseases Work Group (2021) KDIGO 2021 clinical practice guideline for the management of glomerular diseases. Kidney Int 100:S1–S276. 10.1016/j.kint.2021.05.021 34556256

[29] Trautmann A Boyer O Hodson E Bagga A Gipson DS Samuel S. IPNA clinical practice recommendations for the diagnosis and management of children with steroid-sensitive nephrotic syndrome. Pediatr Nephrol. (2023) 38:877–919. 10.1007/s00467-022-05739-3 36269406 PMC9589698

[30] Fabri D Belangero VMS Annichino-Bizzacchi JM Arruda VR. Inherited risk factors for thrombophilia in children with nephrotic syndrome. Euro J Pediatr. (1998) 157:939–42. 10.1007/s004310050972 9835442

[31] Van Ommen CH Nowak-Göttl U. Inherited thrombophilia in pediatric venous thromboembolic disease: why and who to test. Front Pediatr. (2017) 5:50. 10.3389/fped.2017.00050 28352625 PMC5348488

[32] Biss TT Brandão LR Kahr WHA Chan AKC Williams S. Clinical probability score and D-dimer estimation lack utility in the diagnosis of childhood pulmonary embolism. J Thromb Haemost. (2009) 7:1633–8. 10.1111/j.1538-7836.2009.03572.x 19682234

[33] Witt DM Nieuwlaat R Clark NP Ansell J Holbrook A Skov J. American society of hematology 2018 guidelines for management of venous thromboembolism: optimal management of anticoagulation therapy. Blood Adv. (2018) 2:3257–91. 10.1182/bloodadvances.2018024893 30482765 PMC6258922

[34] Dadgar K Xue Y Chung J Sangar S Bhatt M Chan AKC. Childhood nephrotic syndrome and the clinical profile of thromboembolism: a systematic review and meta-analysis. Pediatr Res. (2023) 93:1463–9. 10.1038/s41390-022-02302-6 36127405

[35] Cohen O Levy-Mendelovich S Ageno W. Rivaroxaban for the treatment of venous thromboembolism in pediatric patients. Expert Rev Cardiovasc Ther. (2020) 18:733–41. 10.1080/14779072.2020.1823218 32935597

